# Breathomics profiling of metabolic pathways affected by major depression: Possibilities and limitations

**DOI:** 10.3389/fpsyt.2022.1061326

**Published:** 2022-12-14

**Authors:** Laila Gbaoui, Melanie Fachet, Marian Lüno, Gabriele Meyer-Lotz, Thomas Frodl, Christoph Hoeschen

**Affiliations:** ^1^Chair of Medical Systems Technology, Institute for Medical Technology, Otto von Guericke University, Magdeburg, Germany; ^2^Department for Psychiatry and Psychotherapy, Medical Faculty, Otto von Guericke University, Magdeburg, Germany; ^3^Department of Psychiatry, Psychotherapy and Psychosomatics, University Hospital, RWTH Aachen, Aachen, Germany

**Keywords:** major depressive disorder, breath gas analysis, volatile organic compounds, proton transfer reaction mass spectrometry, metabolomics, breathomics, amino acids, gut bacterium

## Abstract

**Background:**

Major depressive disorder (MDD) is one of the most common psychiatric disorders with multifactorial etiologies. Metabolomics has recently emerged as a particularly potential quantitative tool that provides a multi-parametric signature specific to several mechanisms underlying the heterogeneous pathophysiology of MDD. The main purpose of the present study was to investigate possibilities and limitations of breath-based metabolomics, breathomics patterns to discriminate MDD patients from healthy controls (HCs) and identify the altered metabolic pathways in MDD.

**Methods:**

Breath samples were collected in Tedlar bags at awakening, 30 and 60 min after awakening from 26 patients with MDD and 25 HCs. The non-targeted breathomics analysis was carried out by proton transfer reaction mass spectrometry. The univariate analysis was first performed by T-test to rank potential biomarkers. The metabolomic pathway analysis and hierarchical clustering analysis (HCA) were performed to group the significant metabolites involved in the same metabolic pathways or networks. Moreover, a support vector machine (SVM) predictive model was built to identify the potential metabolites in the altered pathways and clusters. The accuracy of the SVM model was evaluated by receiver operating characteristics (ROC) analysis.

**Results:**

A total of 23 differential exhaled breath metabolites were significantly altered in patients with MDD compared with HCs and mapped in five significant metabolic pathways including aminoacyl-tRNA biosynthesis (*p* = 0.0055), branched chain amino acids valine, leucine and isoleucine biosynthesis (*p* = 0.0060), glycolysis and gluconeogenesis (*p* = 0.0067), nicotinate and nicotinamide metabolism (*p* = 0.0213) and pyruvate metabolism (*p* = 0.0440). Moreover, the SVM predictive model showed that butylamine (*p* = 0.0005, p_FDR_=0.0006), 3-methylpyridine (*p* = 0.0002, p_FDR_ = 0.0012), endogenous aliphatic ethanol isotope (*p* = 0.0073, p_FDR_ = 0.0174), valeric acid (*p* = 0.005, p_FDR_ = 0.0162) and isoprene (*p* = 0.038, p_FDR_ = 0.045) were potential metabolites within identified clusters with HCA and altered pathways, and discriminated between patients with MDD and non-depressed ones with high sensitivity (0.88), specificity (0.96) and area under curve of ROC (0.96).

**Conclusion:**

According to the results of this study, the non-targeted breathomics analysis with high-throughput sensitive analytical technologies coupled to advanced computational tools approaches offer completely new insights into peripheral biochemical changes in MDD.

## Introduction

Major depressive disorder (MDD) is one of the most common psychiatric disorders that can drastically affect individual's daily life, social functioning as well as health systems worldwide. It is a complex and heterogeneous disease affecting around 350 million people worldwide (1), the lifetime incidence of depression is more than 12% in men and 20% in women and will become the leading cause of disability worldwide by 2030 (2–5). Even today, it is challenging to diagnose MDD with an objective and quantitative method. Furthermore, only one third of depressed persons receives an adequate treatment. Unfortunately, failure in early diagnosis and treatment of MDD can be associated with a high suicidal tendency rate and relapse. Approximately 50% of 800,000 suicide deaths annually occur within a depressive episode (6) and up to half of patients under therapy relapse despite the progress of antidepressant drugs. Therefore, tremendous efforts have been undertaken during the last few decades, in order to identify reliable diagnostic biomarkers for MDD. Several mechanisms have been associated with MDD, including monoamine deficits, inflammatory, neurodegenerative alterations (3, 7), hypothalamic-pituitary-adrenal (HPA) axis dysfunction (3, 8, 9), gut microbiota dysregulation (10–12) and energy metabolism deficiency (13, 14). However, no single established mechanism can explain all aspects of this multifactorial disorder. The pathophysiology of MDD depends on alterations in a wide range of biological systems interacting with each other and their perturbation can be successfully assessed only by multi-parametric biomarkers signature. In this context, metabolomics coupled with high-throughput analytical technologies and advanced computational tools such as machine learning approaches offer completely new insights into the heterogeneous pathophysiology of MDD. Metabolomics has recently emerged as a particularly potential quantitative tool that provides specific multi-parametric biomarker signatures reflecting alteration in an array of biochemical processes that underpin MDD (15) and the effects of drugs therapies on those biological processes (16). Metabolomics is the end point of the omics cascade and has the potential to sample endogenous and exogenous small molecule metabolites directly. It represents the downstream products from the alterations that occur at the genetic, transcriptional, and translational levels, as well as perturbation in gut microbiome metabolic and global metabolic response of environmental influence.

Former research suggested that MDD is associated with metabolic disturbances and that metabolomic profiling based on different biological matrices such as blood, plasma, urine or feces may have utility in differentiating MDD from bipolar disorder or healthy controls (HCs) (17, 18). Other studies reported that biochemical change during treatment can predict response to antidepressant medication. Some pharmacometabolomic studies reported perturbation in by-products of tricarboxylic acid cycle, urea cycle, amino acids, and lipids in depressed patients exposed to sertraline (19, 20). Mounting metabolomics studies reported alteration in amino acids. In a cross-sectional study, the branched chain amino acids (BCAAs) valine, leucine, and isoleucine were significantly lower in MDD patients compared to HCs (21). In a rat model of depression, L-aspartic acid, L-glutamine, taurine, γ-amino-n-butyric acid and L-α-amino-adipic acid have been reported as possible potential biomarkers for future diagnosis of depression and development of antidepressant (22). Another rat model study suggested that biogenic amino acids were significantly reduced in the hippocampus of stressed rats compared to non-stressed ones (22). Most of these studies, however, employed a targeted metabolomics approach or restricted their analyses to specific biochemical response to drug therapies.

In the present study, we proposed breathomics as one of the newest branches of metabolomics to explore the metabolic change that are related to MDD. Compared to other biomatrices-based metabolomics, breathomics offer practical advantages. Breath samples are suitable for long-term and frequent monitoring of biomarkers related to disease progression, treatment response or treatment resistance, can be monitored in real time and analyzed in a matter of minutes making it rapid and cost-effective. Additionally, breath gas analysis (BGA) offers the possibility of non-target analysis allowing the detection of a multi-parametric signature that can identify a whole array of chemical changes in MDD patients. Further, due to the advanced throughput sensitive mass spectrometry devices, breathomics is rapidly becoming a discovery tool for early screening, diagnostics, disease monitoring and drug metabolism in a wide range of contexts. These include viral (23, 24) and bacterial infectious diseases (25), metabolic conditions such as diabetes (26), breast cancer (27, 28), lung cancer (29, 30), head-neck cancer (31, 32) and neurological and psychiatric disorders (33–35), Asthma (36), physical and mental stress (37, 38). Despite the advanced mass spectrometry devices, BGA is still in its infancy and the methodology still lacks standardization.

The exhaled breath is a complex matrix with more than 3,000 volatile organic compounds (VOCs), arising from exogenous sources (environmental factors / microbiome) or from endogenous biochemical processes taking place in the lung, air way or in other part of the body as well as at different omics levels (Genes, proteins, etc.). Independent of their origin, metabolites can penetrate from the blood stream to the lung lining fluid where they enter the breath by the alveolar gas exchange mechanisms. Depending on their solubility and volatility, metabolites appear in exhaled breath gas or exhaled breath condensate. Therefore, we hypothesized in the present study that exhaled metabolites could mirror the metabolic alterations in MDD and provide a multi-parametric signature specific to several mechanisms underlying the heterogenous pathophysiology of MDD including disrupted metabolic systems, gut microbiome alteration, energy metabolism disbalance, amino acids deficiency, increased inflammation, and oxidative stress in major depression.

In a previous investigation in our workgroup, random forest and logistic regression were performed to identify markers in exhaled breath that differ between MDD patients and controls. Several masses were significantly different between the cohort groups. Among them the masses m/z = 69, 74, 93, and 94 were the potential markers with a high accuracy (39). However, no interplay of the volatile metabolites in pathophysiological pathways was taken in account in this previous study. Thus, within the present study, we performed an advanced non-targeted breathomics profiling from MDD patients and healthy volunteers by proton transfer reaction mass spectrometry (PTR-MS) coupled to machine learning approaches as well as metabolic pathway analysis to investigate the metabolomics pattern changes that occur in patients with MDD and explore their interaction within metabolic pathways or networks.

The main purpose of the present study was to investigate whether the patients with MDD and HCs could be differentiated according to their breathomics patterns and their altered metabolic pathways using PTR-MS as well as the association of the disrupted metabolites with the already hypothesized mechanisms contributing to MDD. The awakening was chosen in the present research as a natural stress stimulus because awakening-induced processes play an important role in the modulation of several biological and activation of stress-responsive systems such as hypothalamic-pituitary-adrenal (HPA) axis.

To our knowledge, this is the first breathomics study to explore alteration of biochemical patterns and metabolic pathways in patients with MDD relative to HCs using PTR-MS.

## Methods and materials

### Study population

A total of 26 patients with MDD were recruited at the Clinic for Psychiatry and Psychology, Medical Faculty, Otto-von-Guericke University in Magdeburg, Germany. Patients were evaluated according to diagnostical and statistical manual of mental disorders (DSM-IV). Exclusion criterion of MDD patients included a known alcohol or drug dependency, neurological disorders and diseases affecting the brain function. The control group included age, sex and BMI matched 25 healthy subjects between 20 and 60 years without psychiatric or endocrinologic diagnosis, normotensive, non-diabetics, not addicted to tobacco and showed no evidence of any acute or chronic infection. HCs were recruited through local advertisements. The demographical and clinical characteristics of MDD and HCs are listed in Table 1.

**Table 1:** Demographical and clinical characteristics of patients with MDD and HCs.

**Characteristics**	**HCs (*n* = 25)**	**MDD (*n* = 26)**	***p*-value**
Gender (w/m)	13/12	16/10	0.49
Age (years)	34.40 ± 8.15	38.04 ± 12.90	0.24
BMI (kg/m^2^)	24.67 ± 3.96	27.36 ± 7.99	0.21
Education (years)	12.04 ± 3.20	12.15 ± 2.60	0.89
Smoking	0	8	0.003
Alcohol drinking	5	5	0.95
BDI	1.72 ± 3.80	32.56 ± 10.79	1.27 x 10^−19^
HAMD-17	0.12 ± 0.44	17.2 ± 4.9	5.66 x 10^−25^
Medication	n.a		n.a
None		7	
SSRI		7	
SNRI		4	
NASSA		5	
Others		3	

HAMD, Hamilton Depression Rating Scale; BDI, Beck Depression Inventory-II; SSRI, serotonin reuptake inhibitors; SNRI, serotonin and noradrenaline reuptake inhibitors; NASSA, noradrenaline and selective serotonergic antidepressants; others, valdoxane, nortiptiline, and bupropione.

The study protocol was approved by the Institutional Review Board of the Otto-von-Guericke University, Magdeburg. All procedures used in the study were conducted as per international ethical standards. Written informed consent was obtained from all participants after they had received a full explanation of the study procedures.

### Clinical data assessment

Depression severity assessments were conducted using the self-rating Beck Depression Inventory-II (BDI-II) (40), and the observer-rating scale 17-items Hamilton Rating Scale for Depression (HAMD-17) (41). BDI-II is one of the most commonly used scale to assess severity of depression experienced during the past 2 weeks. It consists of 21 questions that measure cognitive, affective and somatic components of depression. The total values of the BDI-II range between 0 and a maximum of 63 points. Interpretation of the total score varies according to different recommendations. BDI-II values below 13 points are regarded as no or minimal depressive symptoms. Values between 14 and 19 points indicate a mild expression of depressive symptoms, values between 20 and 28 points a moderate severity and scores between 29 and 63 are regarded as evidence of severe depressive disorder. HAMD-17 is the most widely used clinician-administered depression assessment scale. It is a multiple-choice questionnaire with 17 questions aiming to detecting core symptoms of depression, including depressed mood, loss of interest, feeling of guilt, psychomotor retardation, insomnia, weight change, suicidal tentation, and impairment of functioning. The total score can range from 0 to 52. Interpretation of the total score varies according to different recommendations; e.g., scores below 7 are regarded as normal or remission; values between 8 and 13 indicate mild depression, scores between 14 and 18 moderate depression and values more than 19 are regarded as severe depression.

### Breath sample collection

After an overnight fasting, three mixed breath samples per volunteer were collected in 3L-Tedlar bags (SKC Inc. Eighthy Four, PA) directly after awakening, 30 and 60 min after awakening. The sampling device and its accuracy are described in detail elsewhere (42, 43). Subjects were carefully instructed to refrain from drinks, consumption of coffee or alcohol, brushing their teeth, or smoking before breath sampling in order to minimize the impact of exogenous VOCs on the concentration of the exhaled gas compounds. Seated volunteers breathed out normally up to complete filling 80% of Tadlar bags. Additionally, no reusable sampling bags were used in this study to avoid samples contamination with old VOCs. The breath samples were processed at the Hospital Clinic for Psychiatry and Psychotherapy Otto-von-Guericke University, Magdeburg after the breath gas collection in order to avoid loss of gas compounds in the sampling bags.

### Breathomics profiling by PTR-MS

The breathomics profiling was performed with high sensitivity quadrupole PTR-MS apparatus produced by IONICON Analytic GmbH, Innsbruck. PTR-MS operated at standard conditions: drift tube voltage: 600 V, drift tube pressure: 2.0 mbar, drift tube temperature: 60°C. The hydronium ion H3O^+^ was used as reagent ion in all measurements. A detailed description of the instrument is reported in literature (44). Briefly, PTR-MS is a chemical ionization mass spectrometry technique based on proton transfer reactions from reagent ion H_3_O^+^ to gaseous organic compounds *R*:


(1) $$\begin{array}{c} H_{3}O^{+}+R\to RH^{+}+H_{2}O \end{array}$$

On each collision with H_3_O^+^ ion, the organic molecules R with higher proton affinity than water is ionized by transfer of the proton H^+^. The produced ions (RH^+^) in this reaction are subsequently mass analyzed in a quadrupole mass spectrometer and detected by a secondary electron multiplier/pulse counting system in order to determine the concentration of this volatile compound in the breath sample. No time-consuming pre-concentration procedures are required by PTR-MS. This technique is also suited for real time and multiple measurements. However, PTR-MS quadrupole systems can not differentiate between substances contributing to the same mass. PTR-MS time of flight (PTR-MS TOF) was used parallel to PTR-MS quadrupole (PTR-QMS) in six patients to identify the overlapped compounds. Moreover, the tentative identification of the detected VOCs with PTR-QMS was based on previous works using PTR-MS and the chemical library created using different trace gases detected by PTR-MS and selected-ion flow-tube mass spectrometry (SIFT-MS) using the soft ionization with hydronium ions (45). Additionally, the mass calibration and compound identification as well as isotope correction with PTR-MS TOF were performed with the interface of the software PTR-MS Viewer (version 3.3, IONICON Analytic GmbH, Innsbruck).

### Statistical analysis

All data in the present study was evaluated using the Statistical and Machine Learning Toolbox in MATLAB. The statistical difference in study population characteristics was determined using independent sample *t*-test and Fischer's exact test. In a pre-processing step, the concentration of raw data was averaged over several spectra belonging to the same sample to reduce the background noise, centered, scaled, non-linear transformed using the Log-transformation. Missing values that exist in more than 20% of samples were removed and missing values that exist in < 80% of samples were corrected with a determined small value such as 2 (46). The distribution of the data was investigated using Kolmogorov-Smirnov test. Additionally, the effect size for group differences between MDD patients and HCs was calculated by Cohen's D approach based on the results of this study, and the power analysis was performed using the G-Power tool (http://www.gpower.hhu.de) to estimate the required population size for future validation (47).

To investigate the metabolomic change in MDD patient, we adopted a several-pronged approach. Univariate analysis was first carried out to obtain an overview and a rough ranking of potentially altered VOCs in MDD patients relative to HCs and to establish features applicable to unsupervised and supervised multivariate approaches in subsequent analysis. Student *t*-test on the log-transformed data was carried out to discriminate between the cohorts' groups. The VOCs with *p*-value lower than 0.05 were considered statistically significant. To account for multiple testing, *p*-values were adjusted according to Benjamin Hochberg approach to control the false discovery rate (FDR). The exogenous compounds related to smoking and VOCs available in lower than 70% of samples were excluded.

In order to investigate the metabolites with similar response to awakening stress that could be involved in same pathways or networks, we focused on identifying clusters of significant VOCs that showed similar alteration from baseline to 60 min after awakening as response to awakening stress. In a first step, a matrix with the changes of metabolites concentrations in logarithmic scale from baseline to 60 min was created and the correlation matrix between these alterations was calculated by Spearman correlation. In a further step, the hierarchical clustering analysis (HCA) was applied on the matrix of Spearman's correlation coefficients (48). HCA is an unsupervised machine learning approach that provide intuitive visualization of the data sets/clusters in order to understand the redundancies of the chemical compounds and to identify clusters of volatile metabolites that could be involved in the same metabolic pathways or biological networks.

Additionally, to examine metabolomics changes manifested in MDD patients more comprehensively, we investigated chemical changes occurring in metabolic pathways and networks using the metabolic pathway enrichment analysis (PEA) and pathway topological analysis (PTA). The significant volatile breath metabolites were mapped into metabolic pathways using the Kyoto Encyclopedia of Genes and Genomes (KEGG) pathways database (49). The PEA compares a list of detected metabolites in each pathway of interest with the metabolites expected to be found in the given pathway. *P*-values for each pathway are calculated based on the hypergeometric distribution (50). PTA was conducted using the relative betweenness centrality and out degree centrality measures to estimate the importance of each metabolite within a specific pathway (51). Pathway impact is the cumulative value of the centrality measures of each matched metabolites normalized by the sum of centrality of all metabolite in a particular pathway.

Furthermore, to investigate the most significant breath metabolites in the identified pathways and clusters, a supervised machine learning analysis was performed by support vector machine (SVM) predictive model. The objective of the SVM algorithm is to find a hyperplane in a high dimensional space that distinctly classifies the data points by maximization of the margin (distance) between the hyperplane and the closest points (52). The receiver operating characteristic (ROC) curves were attained to verify which breath metabolites signature had the highest sensitivity and specificity for a potential MDD diagnosis. The bootstrap technique with 1,000 iterations was used to calculate the bootstrap-corrected AUC and 95% confidence interval (95% CI) of the AUC. Finally, the association between the metabolites of interests and depression severity scores of HAM-D17 and BDI-II was measured by Spearman correlation coefficients.

## Results

### Baseline characteristics of study population

In total, 26 MDD participants (38% men, 62% women), with a mean age of 38.04 ± 12.90 years and BMI of 27.36 ± 7.99 kg/m^2^ were included in this study. The prevalence of mild, moderate and severe depression according to HAMD-17 was 50, 38, and 12%, respectively. No significant differences in age, gender, BMI, alcohol consumption and education were identified between the cohort groups. Patients with MDD show significant higher depression severity compared to HCs (*p* < 0.001). The smoking status was significantly different between the cohort groups (*p* = 0.003). Demographic, lifestyle and clinical information of the study population is summarized in Table 1.

### Breathomics metabolites identification

**Figure 1:**
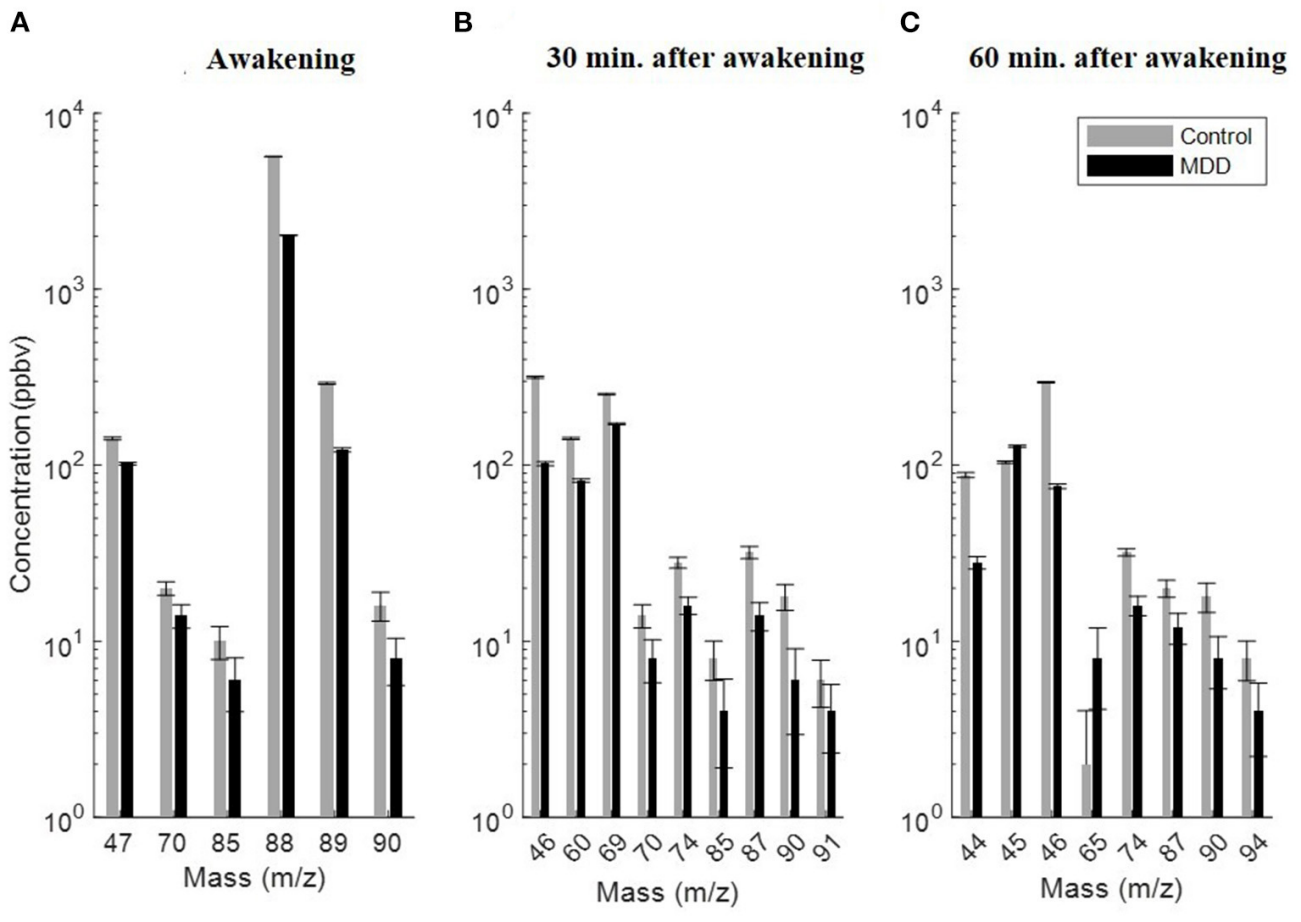
Significant exhaled VOCs discriminating between patients with major depression (black) and healthy controls (gray) after exclusion of smoking VOCs, compounds with inter- and intra-variability and compounds available in lower than 70% of samples. **(A)** At awakening, **(B)** 30 min after awakening, and **(C)** 60 min after awakening. All exhaled VOCs were decreased in the breath of MDD patients except VOCs at acetaldehyde at m/z = 45 and ethanol isotope at m/ = 65. All concentrations of the VOCs are plotted in median and geometric standard deviation.

A total of 132 breath metabolites were identified in 153 samples of a cohort of 51 subjects in range between m/z = 18 to m/z = 150. Out of which, 23 VOCs were statistically different between MDD patients and HCs at level *p* < 0.05. These VOCs belong to various chemical classes, namely alcohols, aldehydes, amino acids, biogenic amines, benzoic acids, hydrocarbons compounds, pyridines, unsaturated hydrocarbon, short chain fatty acids (SCFAs), short chain fatty acids esters (SCFAEs). Table 2 summarizes the significant exhaled VOCs. The significant exhaled metabolites discriminating between Patients with MDD and HCs after exclusion of smoking VOCs and and compounds available in lower than 70% of samples are illustrated in Figure 1. Additionally, the most significant different VOCs between MDD and HC with the univariate analysis (*p* < 0.01) were butylamine at m/z = 74 (*p* = 0.0005, P_FDR_ = 0.0006), methylpyridine at m/z = 94 (*p* = 0.0002, P_FDR_ = 0.0012), endogenous aliphatic ethanol isotope at m/z = 65 (*p* = 0.0073, P_FDR_ = 0.0174) and valeric acid (interference with lysine) at m/z = 85 (*p* = 0.005, P_FDR_ = 0.0162), where P_FDR_ the adjusted *p*-value for multi-testing.

**Table 2:** Significant breath metabolites that discriminate between patients with MDD and HCs at significance level *p* < 0.05.

**Ion m/z**	**Tentative identification**	**Class**	***p***	**p_FDR_**	**Regulation**
42	Acetonitrile	Aromatic hydrocarbons	0.009	0.035	Up
44	Ethenamine	Amine	0.026	0.037	Down
45	Acetaldehyde	Aldehyde	0.036	0.040	Up
46	Ethylamine	Amine	**0.004**	0.012	Down
47^p^ /65*	Ethanol	Alcohol	0.040/**0.007**	0.047/0.017	Down/up
60	Acetate Trimethylamine^int^	SCFAs Amine	0.047	0.048	Down
69 / 70*	Isoprene	unsaturated hydrocarbons	0.038/0.020	0.045/0.038	Down/down
74	n-Butanamine	Amine	**0.0005**	**0.0006**	Down
93*(75^p^)	Propionic acid (Benzene^int^)	SCFAs	**0.0004**	**0.0048**	Up
79* (61^p^)	Acetic acid (Benzene^int^)	SCFAs	0.024	0.039	Up
85^a^ (103^p^) 85^a^ (147^p^)	Valeric acid isotope Lysine isotope^int^	SCFAs Amino acid	**0.005**	0.016	Down
87	2,2-Dimethylbutane 2,3-Butandione^int^	Alkane Alcohol	0.015	0.038	Down
88	n-Butyrate	SCFAs	0.041	0.041	Down
89	(iso)Butyric acid Methyl propionate	SCFA SCFAEs	0.029	0.035	Down
90	Alanine	Amino acid	0.025	0.037	Down
91	2,3-Butandiol	Alcohol	0.048	0.048	Down
94	3-Methylpyridine	Pyridine	**0.0002**	**0.001**	Down
102	2-Methylbutyrate 1-Butanamine, N,N- dimethyl	SCFAEs Amine	0.0232	0.040	Down
106^p^ (88^a^)	Serine	Amino Acid	0.040	0.041	Down (down)
116	1-Propane-amine, N,N-dimethyl	Amine	0.037	0.044	Down
123	Nicotinamide	Pyridine	0.0269	0.0336	Down
132 /86^a^	(Iso)leucine	Amino Acid	0.0384/0.033	0.0477/0.039	Down
138	Anthranilic acid	Benzoic acid	0.038	0.047	Up

^**a**^Abundant at this mass; ^**int**^interference; ^p^precursor VOCs; ^*^fragment or isotope; SCFAs, short chain fatty acids; SCFAEs, short chain fatty acid esters.

The p_FDR_ values are the adjusted *p*-values for multi-testing. The bold value indicate the most significant metabolites (*p* < 0.01).

**Figure 2:**
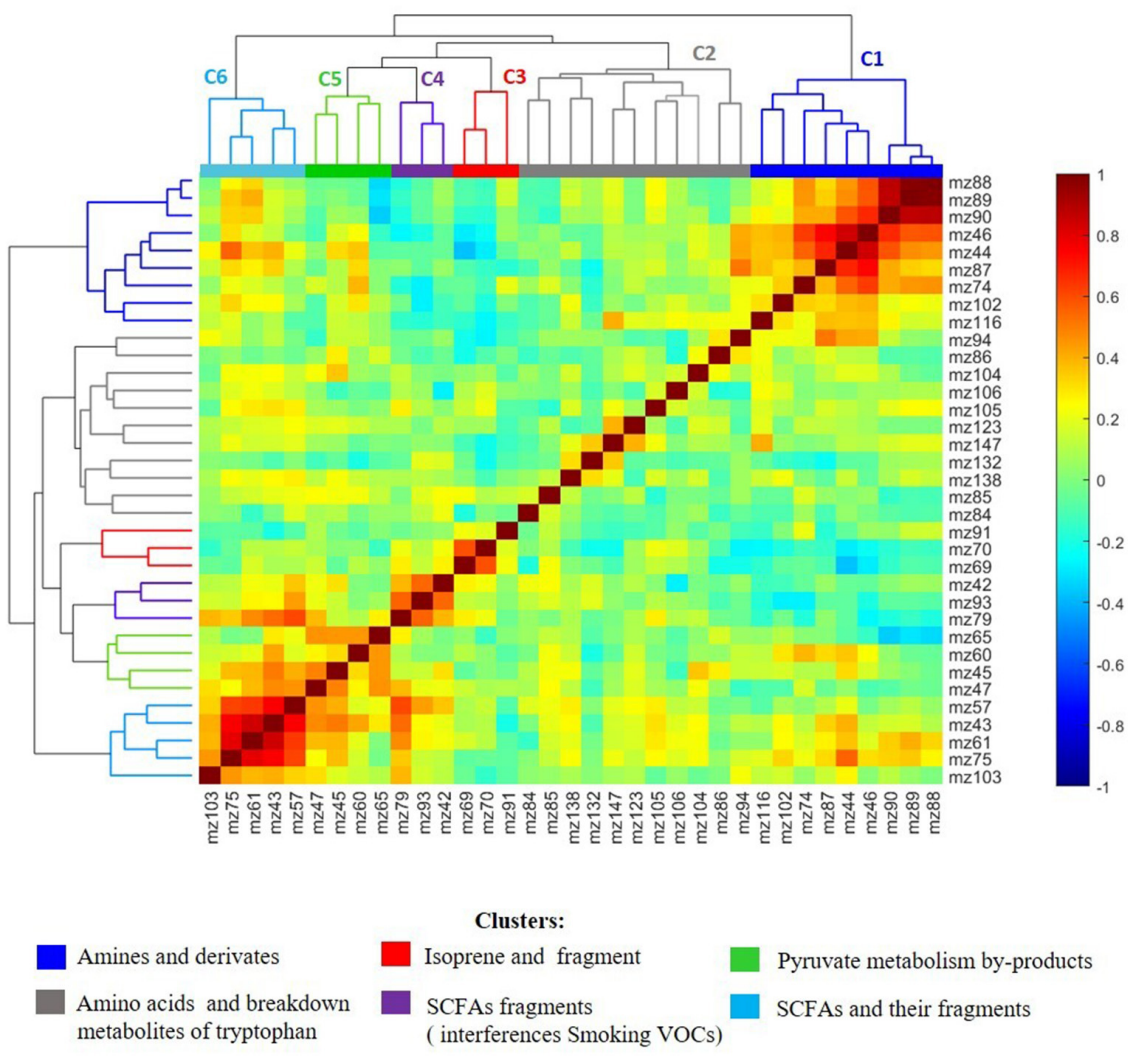
Heatmap visualization and hierarchical clustering analysis of Pearson's distance between significant altered breath metabolites of MDD patients compared to healthy controls (*p* < 0.05) from awakening to 60 min after awakening. The HCA showed the presence of six clusters of breath metabolites in which the member VOCs showed statistically significant correlation among each other in their perturbation from baseline to 60 min after awakening. The clusters are primarily predominated with the same chemical class or metabolites involved in the same pathway/network. The cluster C1 **(dark blue)** contained mainly amines, C2 **(gray)** amino acids and breakdown metabolites of amino acid tryptophan, C3 **(red)** isoprene and its fragment at m/z = 70, C4 **(violet)** fragments of SCFA that are overlapped with aromatic smoking VOCs, C5 **(green)** metabolites involved in pyruvate metabolism and C6 **(light blue)** included SCFAs and their fragments. The metabolites in C2 showed lower correlation with each other due to the intra- and inter-variability of amino acids in exhaled bread using PTR-MS.

The significant exhaled metabolites showed lower responsiveness to the awakening stress and were decreased in the breath of patients with MDD compared with HCs except ethanol, acetaldehyde and anthranilic acid. Further, levels of all exhaled VOCs were affected by smoking habits. The concentration of all VOCs was decreased but not significantly in the breath of MDD smoker compared to non-smoker patients except isoprene at m/z = 69, isoprene fragment at m/z70, and lysine/valeric acid fragment at m/z = 85 that were relatively increased in smoker MDD patients compared to non-smoker ones. Additionally, no significant impact of the medication on the VOCs concentration was observed. Moreover, as shown in Tables 2, 3 and Figure 2, the composition of altered metabolites in MDD breath was clearly dominated by volatile amino acids (AAs) and gut microbiota metabolic byproducts such SCFAs and acetaldehyde as well VOCs involved in the metabolism of AAs by intestinal tract gut microbiota including intermediate metabolites of the essential amino acid tryptophan and biogenic amines.

**Table 3:** Clusters of differential breath metabolites with similar responsiveness to awakening stress from baseline to 60 min after awakening clustered by HCA and the corresponding primarily dominant chemical class or probable pathway.

**Cluster**	**m/z**	**Metabolites**	**Class / pathway**
Cluster #1	44 46 74 87 102 116.14	Ethenamine Ethylamine 1-Butanamine 2,2-Dimethylbutane 1-Butanamine, N,N-dimethyl 1-Propane-amine, N,N-dimethyl	Biogenic amines: Microbial decarboxylation products of amino acids
	88 89 90	n-butyrate (Iso)Butyric acid Alanine	SCFAs Amino acids
Cluster #2	147^p^, 84*,85^*a^ 106^p^, 88* 132^p^, 86^*a^	Lysine Serine Iso(Leucine)	Amino acids
	123 138 94	Nicotinamide Anthranilic 3-Methylpyridine (precursor of nicotinamide)	Breakdown metabolites of tryptophan metabolism in Tryptophan-Nicotinic pathway
Cluster #3	69^p^/70* 91	Isoprene 2,3-Butandiol	mevalonate pathway
Cluster #4	42 79* 93*	Acetonitrile Acetic acid (Benzene^int^) Propionic acid (Toluene^int^)	SCFAs fragments (interference with aromatic smoking VOCs)
Cluster #5	47^p^/65* 45 60	Ethanol Acetaldehyde Acetate	Pyruvate metabolism Glycolysis
Cluster #6	61^p^, 43* 75^p^, 57* 103^p^	Acetic acid Propionic acid Valeric acid	SCFAs

^a^VOCs are more abundant at this mass; ^*^fragment/isotope of VOCs; ^int^interference; ^p^precursor VOC (main Compound).

Additionally, based on the metabolites of interest in this study, an effect size of d = 1.26 for group differences between MDD patients and HCs using Cohen's D method was calculated. Assuming a middle effect size of d = 0.6, an error of 0.05 and a statistical power 0.95 to be enough, 60 subjects per group are required for the difference between patients with MDD and HCs. In order to investigate the effect of medication, nutrition, smoking and hormonal status in females on the VOCs, we aim to recruit in total 80 patients at baseline and 80 HCs in the test sample.

### Hierarchical cluster analysis of metabolomic profile

As illustrated in Figure 2, HCA revealed the presence of seven metabolite modules in which the respective metabolites showed statistically significant correlation (*p* < 0.05) among each other in their perturbation patterns from baseline to 60 min after awakening. The detected clusters were represented by metabolites belonging primarily to the same biochemical class or involved in a specific metabolic network/pathway and have functional relationships with each other. Cluster 1 (C1) included mainly biogenic amines produced by microbial decarboxylation of amino acids. Ethylamine and butylamine are decarboxylase products of non-essential alanine and norvaline, respectively and their derivates including 1-Butanamine,N-N-dimethyl and 1-Propane-amine, N,N-dimethyl as derivate from propanamine that is decarboxylate product of the amino acid alpha-aminobutyrate (16). Cluster 2 (C2) contained mainly essential volatile amino acids and intermediate metabolites involved in the metabolism of the essential amino acid tryptophan *via* tryptophan-nicotinate pathway. Cluster 3 (C3) encompassed isoprene and its fragment at m/z = 70. The association of the compound at m/z = 91 to isoprene cluster is not clear. Cluster 4 (C4) included primarily fragments of acetic acid and propionic acid that showed strong interference with the smoking VOCs benzene and toluene at m/z = 79 and m/z = 93 respectively. Cluster 5 (C5) included volatiles involved in pyruvate metabolism and cluster 6 (C6) included SCFAs propionic, acetic and valeric acids and their isotopes. The presence of (iso)butyrate at m/z = 89 in C1 and not in C6 could be indicator that this SCFA was not produced by anaerobic fermentation of fiber, but rather by bacterial fermentation of proteinaceous material such as amino acids. The results of the inter-correlation between the metabolites and hierarchical clustering are visualized in a heat map and dendrogram cluster tree in Figure 2 and clusters details are presented in Table 3.

### Metabolomic pathway analysis

**Figure 3:**
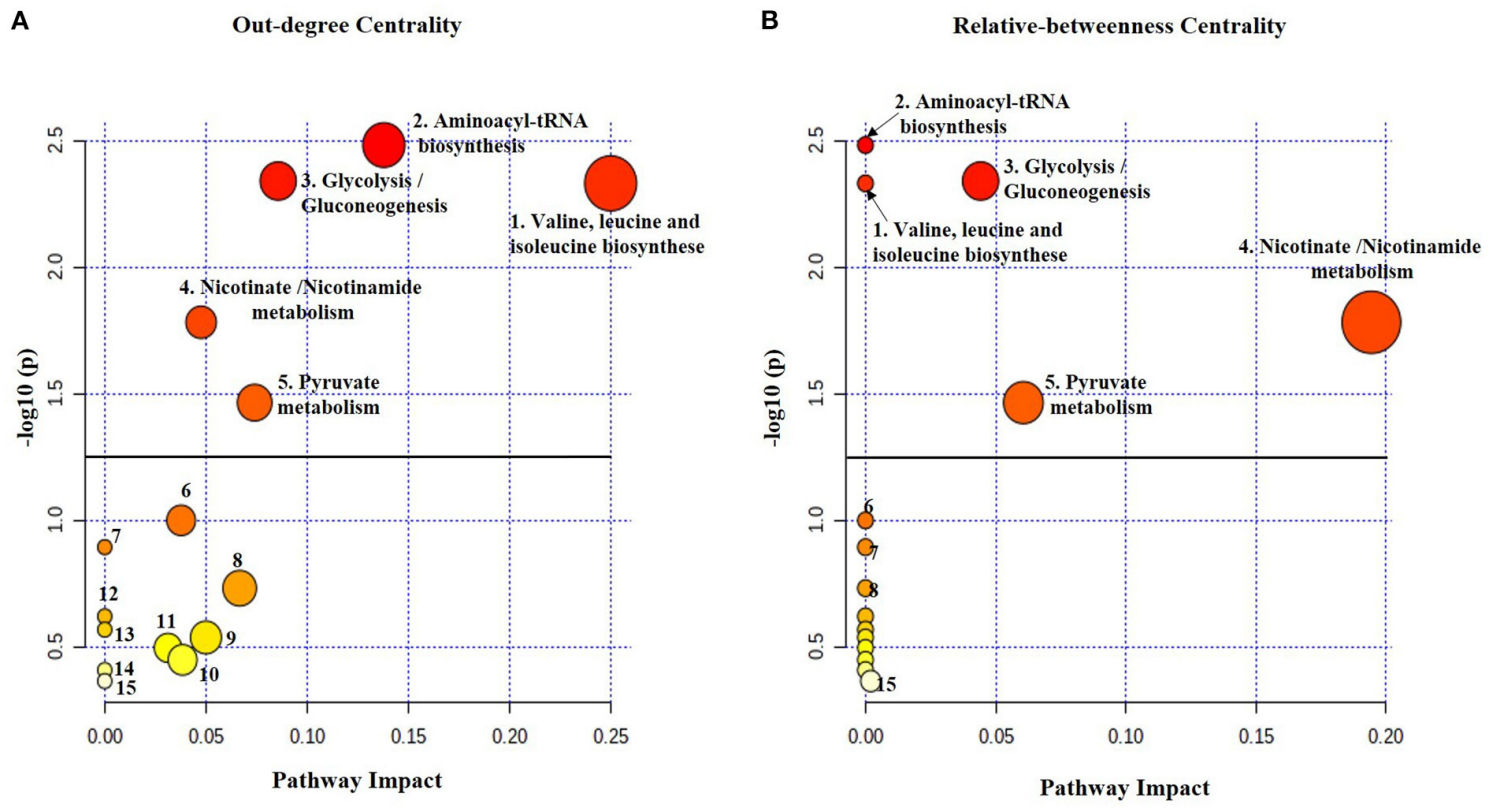
The metabolome view map of altered metabolic pathways identified in breath samples from MDD patients compared with healthy controls conducted by MetaboAnalyst tool. The significant pathways with the enrichment analysis are (1) Valine, leucine and isoleucine biosynthesis, (2) Aminoacyl-tRNA biosynthesis, (3) Glycolysis and gluconeogenesis, (4) Nicotinate and nicotinamide and (5) Pyruvate metabolism. The topological pathway analysis shows different impact factors of the significant altered pathways using **(A)** out-degree centrality and **(B)** relative betweenness centrality. The matched nodes show varied heat map colors and is based on p-value while the node radius is determined based on the pathway impact values. The bold lines indicate the significance level of a *p*-value = 0.05. The pathways under the bold line are probable altered pathways but not significant (6) Valine, leucine and isoleucine degradation, (7) Biotin metabolism, (8) Butanoate metabolism, (9) Lysine degradation, (10) Glyoxylate and dicarboxylate, (11) alanine, aspartate and glutamate metabolism, (12) Selenocompound metabolism, (13) Propanoate metabolism, (14) Glycerophospholipid metabolism and (15) Tryptophan metabolism.

The metabolic pathway analysis (PA) revealed that 16 pathways could be affected in MDD patients compared with controls, as shown in Figure 3. However, the most significant pathways (*p* < 0.05) were (1) aminoacyl-tRNA biosynthesis; (2) BCAAs valine, leucine, isoleucine biosynthesis; (3) glycolysis/gluconeogenesis; (4) nicotinate and nicotinamide metabolism as well as (5) pyruvate metabolism at level *p* = 0.0033, 0.0047, 0.0046, 0.0165, 0.0343, respectively. The pathway impact changed depending on the centrality measure used by TPA. Aminoacyl-tRNA biosynthesis and branched chain amino acids (BCAAs) valine, leucine, isoleucine biosynthesis showed the highest impact using out degree centrality, whereas nicotinate/nicotinamide metabolism and pyruvate metabolism showed the highest impact using relative betweenness centrality. A detailed summary of the most relevant pathways is given in Table 4.

**Table 4:** Significant altered metabolic pathways in patients with MDD compared with healthy subjects (*p* < 0.05) using pathway enrichment analysis (PEA) and pathway topological analysis (PTA).

**Pathway name**	**Match status**	**Detected metabolites**	**PEA**		**PTA**	
			***p*-value**	**-Log_10_(p)**	**OD**	**RB**
Aminoacyl-tRNA biosynthesis	4/48	Alanine, lysine, isoleucine, leucine	0.0033	2.4827	0.1379	0
Valine, leucine and isoleucine biosynthesis	2/8	Leucine, isoleucine	0.0047	2.3314	0.25	0
Glycolysis/Gluconeogenesis	3/26	Acetaldehyde, Ethanol, Acetate	0.0046	2.3406	0.0857	0.0442
Nicotinate /nicotinamide metabolism	2/15	Nicotinamide, nicotinate	0.0165	1.7822	0.0476	0.1943
Pyruvate metabolism	2/22	Acetaldehyde, Acetate	0.0343	1.4645	0.0741	0.0607

The pathway analysis was based on the altered breathomics metabolites. PTA was performed using out-of-degree (OD) and relative betweenness (RB) centrality measures to calculate the impact factor of the pathway.

### Supervised multivariate analysis

**Figure 4:**
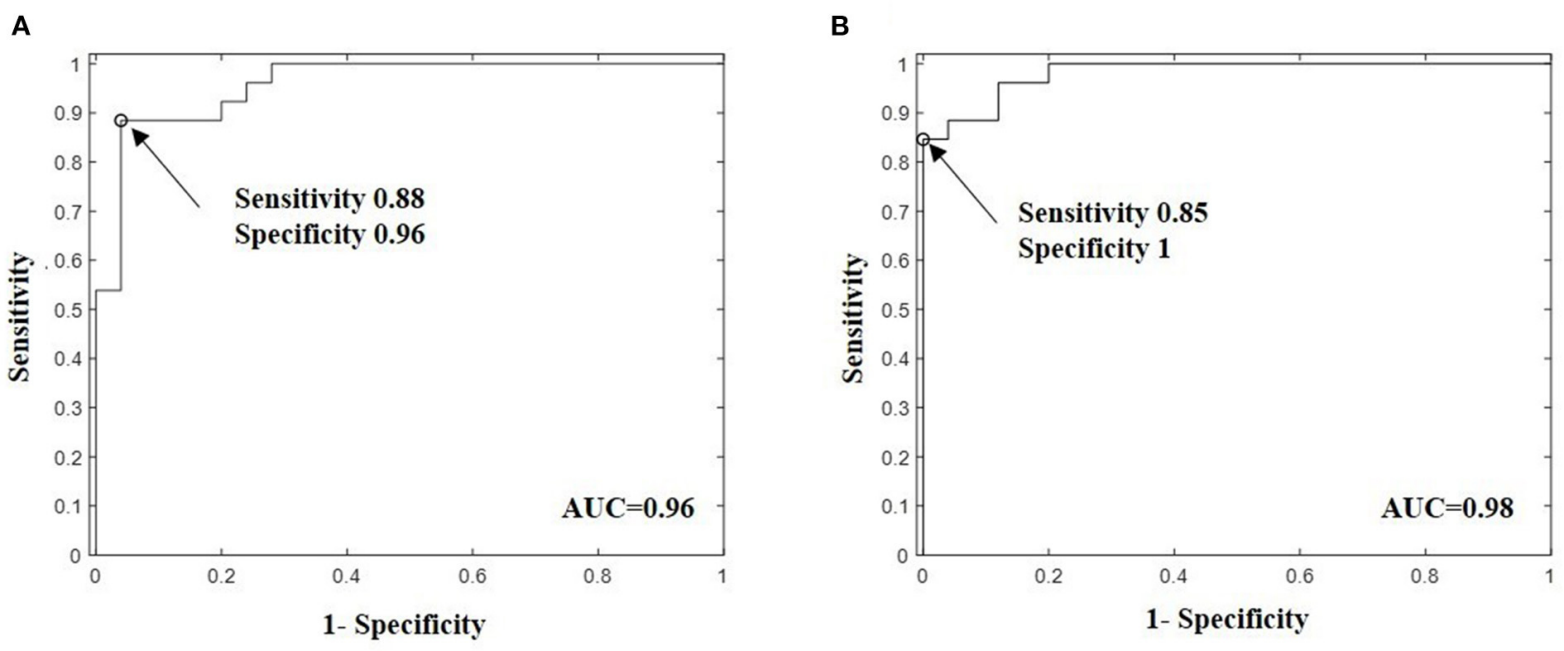
Bootstrap adjusted receiver operating characteristic (ROC) curves for support vector machine (SVM) predictive model built with volatile breath metabolites with highest ability to discriminate MDD patients against healthy persons. **(A)** SVM model with the fingerprint ethanol, isoprene, butylamine, lysine/valeric acid, and 3-methylpyridine showed a sensitivity of 0.88, specificity 0.96, and bootstrap adjusted area under curve AUC of 0.96 (95% CI: 0.8606–0.9903) and **(B)** SVM model with the signature ethanol, isoprene, butylamine, valeric acid and 3-Methylpyridine, and propionic acid fragment showed higher bootstrapped-corrected AUC of 0.98 (95% CI: 0.9318 −0.9971) and specificity of 1 but lower sensitivity 0.88. The Bootstrap technique with 1000 iterations was used to calculated the adjusted AUC and 95% confidence intervals. The arrows in the panels indicates the point on the ROC curve with the optimal sensitivity and specificity.

According to the ROC analysis, the predictive model with SVM showed that the combination of butylamine, 3-methylpyridine, isoprene, ethanol and valeric acid defined the best breathomics signature with a bootstrap corrected AUC value of 0.96, bootstrap adjusted 95% CI of AUC 0.861–0.990, specificity 0.96 and sensitivity 0.88 for class separation between study cohorts, as shown in Figure 4. Interestingly, butylamine, 3-methylpyridine, isoprene, ethanol and valeric acid represented the most significant VOCs within clusters of biogenic amines (C1), amino acids and tryptophan metabolism (C2), isoprene (C3), pyruvate (C5), and SCFAs (C6) revealed by HCA, respectively. Adding propionic or acetic acid fragments from C4 that showed overlapping with the aromatic smoking compounds toluene and benzene at m/z = 93 and m/z = 79, respectively, we found better bootstrap-corrected AUC value of 0.98 (95% CI: 0.931–0.997) and specificity 1 but lower sensitivity 0.84.

### Metabolite association to depression severity

To evaluate whether the volatile metabolites are related to the severity of depressive symptoms, we performed Spearman correlation analysis using a total score of BDI-II and HAMD-17 and concentration of breath metabolites from baseline to 60 min after awakening. We found a significant correlation between HAMD's total score and exhaled valeric acid (*r* = −0.42, *p* = 0.04) at awakening, butylamine (*r* = 0.38, *p* = 0.05), acetaldehyde (*r* = −0.71, *p* = 0.05), butyric acid (*r* = 0.44, *p* = 0.03), n-butyrate (*r* = 0.43, *p* = 0.03) at 30 min after awakening, as well as a significant correlation between BDI-II total score and butylamine (*r* = −0.040, *p* = 0.045). However, after correction for multiple testing of the correlations, they did not survive this adjustment.

## Discussion

There is an unmet clinical need for understanding the heterogeneous pathological events occurring in MDD and for objective markers to monitor disease progression and treatment response. In this study, we used a non-targeted breath-based metabolomics approach to identify biochemical changes that occur in patients with MDD. Our results showed that five metabolic pathways were significantly affected and enriched in MDD patients. In total, 23 differential metabolites were assigned in these pathways. Among these metabolites, isoprene, methylpyridine, ethanol, lysine/valeric acid, and butylamine were identified by SVM predictive model as potential metabolite signature discriminating between MDD patients and HCs with a high AUC of 0.96, sensitivity of 0.96, and specificity of 0.88. Whereas, the other volatiles have been already identified as potential metabolite fingerprints of major depression in previous bio-fluids and fecal metabolomics works. Additionally, our findings showed that breath gas analysis provided a wide range of metabolites involved in several mechanisms that have been already hypothesized in former works to contribute to the pathophysiology of MDD including alteration of gut microbiota (11), dysregulation of amino acids that are generally free in plasma and their breakdown products (21, 53), alteration of intermediate volatiles involved in the metabolism of the slightly volatile amino acid tryptophan, increased inflammation, elevated oxidative and nitrosative stress (54, 55) and energy metabolism imbalance (14). Furthermore, the significant exhaled chemical compounds in the breath of MDD patients belong mainly to three groups; (1) volatile amino acids, (2) volatile gut microbiota by-products produced *via* several fermentation pathways (Pyruvate breakdown metabolites, SCFAs, 2,3-butanediol, 2,3-butandione) and (3) volatile gut microbiota compounds involved in amino acid metabolisms such as microbial decarboxylation products of amino acids such as butylamine, and microbial deamination of amino acids into fatty acids.

The alteration of gut microbiota derived metabolites in depressed persons in this study was in line with previous biomatrices based metabolomics works. A growing body of literature supports associations between major depression and alteration of gut microbiota as well its derived metabolites in intestinal tract (11, 12, 56). Fecal and blood based metabolomics studies demonstrated correlation between bacterial fingerprints with depression (57). Decreases in the diversity and abundance of the gut microbiota was linked to depression and psychological stress in human and animal models exposed to mild, chronic or prolonged stressors (58, 59). Several bacterial metabolic by-products are often considerate as key mediators of gut-brain crosstalk (2, 60). SCFAs acting within intestinal endocrine cells stimulate the production of neuroactive molecules such as serotonin and γ-aminobutyric acid (61). Accumulating evidence supported by several studies in humans and animals reported that alteration of SCFAs is linked to behavioral and neurological pathologies, such as depression (57), Alzheimer's, Parkinson's diseases and autism spectrum disorders (60) as well metabolic disorders such as type 2 diabetes and obesity (62). Moreover, SCFAs play a very important role in regulating energy metabolism and energy supply as well as maintaining the homeostasis of the intestinal tract (63), regulating mitochondrial function (64), regulation of inflammatory processes, emotional state and cognition through the gut-brain axis (65, 66). The levels of exhaled butyric (116 ± 3.10 vs. 169 ± 3.54), acetic (124 ± 2.44 vs. 146 ± 2.31), and valeric acid (4 ± 2.07 vs. 8 ± 2.16) were decreased in MDD patients in comparison to HCs. This is in accordance with previous metabolites studies based on other bio-matrices (66) and animal models (67). Propionic acid was found to be lower in MDD patients relative to HCs (28 ± 3.33 vs. 30 ± 2.2) but not significant. However, its level was significantly decreased in smoker MDD patients (*p* = 0.031) and increased in non-smoker relative to HCs. The alteration of propionic acid concentration in major depression have been confirmed in several researches, however the results concerning the concentration were conflicting. Moreover, SCFAs are mainly absorbed by colonocytes. Only a part of colon-derived acetate, propionate and butyrate reaches the systemic circulation and other tissues. In this context, it is important to note that detected SCFAs in the breath are related to blood SCFAs concentration and cannot be used as a proxy of the production in the colon. Despite the small part of free SCFAs in systemic circulation, the impact of these metabolites on the health was reported in several studies (68, 69). Additional dysregulated bacterial downstream metabolites produced by the significantly altered glycolysis and also involved in pyruvate metabolism in this study are acetaldehyde and endogenous ethanol (EE) at m/z = 65. The levels of these VOCs were significantly increased in the breath of patients with MDD compared to non-depressed ones. EE is constantly formed from acetaldehyde within the human body through metabolic processes. Generally, the concentration of EE in blood of healthy persons is very low. However, intestinal dysbiosis increases EE production, which also affects gut permeability, disrupting intestinal tight junctions and allowing the endotoxins and ethanol to reach the liver and activate pro-inflammatory response. Additionally, EE increases the activity of enzyme cytochrome P450 2E1 which catalyzes the oxidation of ethanol and promotes oxidative and nitrosative stress (70). Increased EE has been reported in several conditions and metabolic disorders such as diabetes mellitus (60), non-alcoholic fatty liver disease (71) and obesity (72). The increased alveolar elimination of endogenous ethanol in exhaled breath of patients with MDD can be attributed to both its increased production and decreased metabolic breakdown associated with altered intestinal microbial activity that is well-known in psychological and chronical stress. Former researchers reported unusually high concentrations of EE in blood and cerebrospinal fluid from hospitalized patients suffering from various psychiatric disorders (73). On the other hand, increased ethanol production was linked to significant abundance of *Candida albicans* in antibiotic-disrupted bacterial community of the murine cecum in mice (74). Additionally, the dysregulation of glycolysis and gluconeogenesis in this pilot study is in accordance with previous omics publications. Mounting omics studies suggested a link between pathogenesis of depression and imbalanced energy metabolism (13, 14). MDD patients have generally mitochondrial energy metabolism obstacles and energy imbalance in the brain (14, 75).

The second group of altered metabolites comprised the volatile amino acids. HCA revealed that two of five significant altered clusters (excluding cluster 4 due to interference with smoking VOCs) were linked to amino acids and their intermediate break down products in MDD patients relative to HCs. Additionally, metabolic PA showed that three out of five significant altered pathways are associated with amino acids including aminoacyl-tRNA biosynthesis and BCAAs valine, leucine and isoleucine metabolism as well as nicotinate and nicotinamide metabolism. Increasing evidence suggested that amino acids and their metabolites are basic substrates and regulators in many metabolic pathways. Accumulating evidence showed that dysregulation of amino acids contributed to the pathophysiology of depression (19, 21, 76, 77) and is associated with mental and physical stress in animal models (76, 78). Consistent with these previous metabolomics reports based on different bio-matrices, we found in the present study a wide range of disrupted levels of amino acids in MDD patients including lysine, alanine, proline, serine, cysteine, leucine/isoleucine as well as intermediate metabolites involved in the metabolism of the essential amino acid tryptophan *via* Kynurenine-nicotinic pathway. However, leucine/isoleucine and alanine were the most abundant and significantly reduced in MDD group compared to controls. Lysine and valeric acid fragments were overlapped at m/z = 85 and should be investigated in further works with PTR-TOF. The other amino acids showed a strong intra-variability and inter-variability or were detectable in < 70% of the measured samples. Cysteine was detected in 65% of MDD and was significantly decreased (*p* = 0.037) compared to HCs. Glycine was increased in MDD patients but not statistically significant. These AAs should be investigated in future breathomics studies with more sensitive instrument. García-Gómez and workers have reported that slightly volatile amino acids namely alanine, valine, isoleucine, leucine, glycine, proline, lysine, phenylalanine, and ornithine can be quantified in the human breath condensate in real time using secondary electrospray ionization coupled to high-resolution mass spectrometry and that their concentrations correlate with plasma concentrations. High variation occurred particularly in amino acids with a low plasma concentration (79, 80).

Decreased concentration of BCAAs leucine/isoleucine in depressive patients has been linked to reduced activation of the Mammalian Target of Rapamycin pathway, leading to depressive symptomology and lower mitochondrial energy metabolism that is common in psychoneurological disorders (14, 21). Moreover, pharmacologic studies reported that BCAAs were increased in depressed patients responding to antidepressant medication compared to the treatment failure group (19). Alanine is a non-essential amino acid that occurs in high levels in plasma. It is produced from pyruvate by transamination and can be also synthesized from BCAAs. It is directly involved in gluconeogenesis and regulates glucose and acid metabolism (https://pubchem.ncbi.nlm.nih.gov/), while leucine and isoleucine participate in the production of energy. Alanine provides energy for muscle tissue, brain and central nerve system. Alanine also plays an important role in lymphocyte reproduction, immunity and is an inhibitory neurotransmitter in the brain. The decreased level of the exhaled alanine in MDD patients in this study could be correlated with deficiency of plasma free alanine that is often related to deficiency of BCAAs or pyruvate that is in turn associated with reduced quality of life (https://hmdb.ca). Despite the critical role of alanine in energy balance, immunity and neurotransmitters production, the association between this amine and depression still requires more research.

Lysine is also involved in the significantly altered aminoacyl-tRNA biosynthesis. Its level at precursor m/z = 147 was significantly upregulated in the exhaled breath of MDD patients compared to non-depressed ones (*p* = 0.049) but showed strong intra- and inter-variability. This is may be due to limit of detection at this mass with the used PTR-MS. Lysine was more abundant at m/z = 85 and decreased in the breath of MDD patients analyzed with PTR-MS TOF, but overlapped with valeric acid and should be investigated with PTR-MS TOF in a larger population. A blood-based metabolomics study with animal model exposed to mild and chronic stress reported that a differential metabolites fingerprint including lysine clearly distinguished resilient rats from susceptible rats. Lysine level was found to be significantly reduced in resilient rats (81) and animals exposed to heat stress (78).

The third group of disrupted exhaled VOCs in the breath of MDD patients in the present research included primarily metabolites involved in metabolism of AAs by gut bacterium. Anthranilic acid (AntA), 3-methylpyridine and nicotinamide are metabolites involved in tryptophan metabolism *via* tryptophan-nicotinic pathway. AntA is intermediate metabolite *via* the Kynurenine pathway, whereas 3-methylpyridine and nicotinamide are involved in the significantly altered nicotinic and nicotinamide pathway. Tryptophan metabolism yields the generation of several neuroactive agents within the central nerve system (CNS) including the neurotransmitter serotonin (82). The activation of sympathetic nervous system has been shown to contribute to upregulation of brain tryptophan concentrations after stress-related and immune challenges in experimental animals. HPA may activate tryptophan metabolism using corticosteroids due to awakening stress. AntA was found significantly upregulated in the exhaled breath of all MDD patients with severe depression and in one patient with the highest moderate depression score according to HAMD-17 and BDI-II. No difference was observed between HCs and MDD volunteers with mild depression. This finding may suggest that AntA level could mirror the depression severity and should be investigated in a larger population. Increased AntA levels have been reported in different mental disorders such schizophrenia (83) and MDD (55) as well as metabolic disorders including chronic hepatitis C, rheumatoid arthritis and osteoarthritis (84) and type 1 diabetes (85, 86). A clinical study reported that blood serum level of AntA may predict the onset and progression of clinical depression (55). Interestingly, an increase in AntA concentration was associated with decreased levels of anti-inflammatory factors in high risk MDD subjects. Pawolwski et al. (86) provided first direct evidence of a role for anthranilic acid in the pathogenesis of inflammation-induced MDD (86). Generally, downstream metabolites of the kynurenine pathway and availability of tryptophan play critical role for the functioning of efferent nerve system and CNS and thus in the gut-brain communication. Moreover, Kynurenine pathway was revealed with PA as a probable but not significant altered pathway. This is primarily due to the slight volatility nature of the metabolites involved in this part which require mass range up to m/z = 265, which is beyond the mass range limit of the used PTR-MS instrumentation (~150–200). Only anthranilic acid was detected in this pathway by PTR-MS. A previous study detected 20 low volatile metabolites of this pathway in exhaled human breath condensate using secondary electrospray ionization coupled to high-resolution mass spectrometry (1).

3-methylpyridine is the main precursor to nicotinic acid (NA), also known as B3 or niacin, whose derivates play critical role in energy metabolism in the living cell and DNA repair. NA deficiency is known to manifest various psychiatric symptoms and is associated with Alzheimer, Parkinson, Huntington diseases, cognitive impairment, schizophrenia or depression (87). A cross section study suggested that increased daily dietary vitamin intake including B3 might protect the public against depression. Although the role of this compound in depression remains unclear.

PA was based on the KEGG library that is originally for genome analysis. Some metabolites might not be identified in humans and require manually checking. There are some significant VOCs reported in Table 2 that were not taken into account or not identified in KEGG pathway library and worth further discussion for their associations with MDD status. Isoprene is thought to be a by-product of cholesterol biosynthesis along the mevalonate acid pathway (88). However, its physiological meaning has not yet been established, although it is known that this VOCs has an endogenous origin and it is not produced in the airways. There is evidence of isoprene production being a protective stress response to toxins and temperature change, as seen in plants but not yet validated in humans (89, 90). The reduced level of isoprene in depressed persons may indicate decreased responsiveness to awakening stress compared with non-depressed ones. Downregulated exhaled isoprene levels have been found in several conditions including cystic fibrosis (91) and cancers (92). Furthermore, numerous studies have suggested that exhaled isoprene can be utilized as a non-invasive and quick established biomarker for blood cholesterol alteration (93, 94) that has been associated with various psychiatric disorders including MDD (95). Moreover, cholesterol is required for correct functioning of neurotransmission in the CNS, and low serum total cholesterol level alters the metabolism of the neurotransmitter serotonin which is involved in the energy metabolism and is negatively correlated with depression severity, poor control of aggressive impulses and increased risk of suicides (96, 97). Thus, the downregulation of the exhaled isoprene in depressed participants in comparison to controls in this study could be a marker for decreased serum cholesterol, reduced serotonin function or reduced storage of tryptophan that is a building block to proteins from which serotonin is made.

The main volatile biogenic amines (VBAs) that were found significantly disrupted in MDD patients compared to HCs are ethenamine, ethanamine, butylamine, and trimethylamine. Trimethylamine is the precursor of the trimethylamine N-oxidase (TMAO) that has a negative association with a broad range of diseases including neurological, metabolic and brain disorders (98, 99). Although the link between TMAO levels and neurological disorders has been previously hypothesized (10, 100), its role in the disease etiology has not been fully explored. Additionally, there is convincing evidence suggesting an association between TMAO and inflammation that is well-known to contribute to the pathophysiology of MDD. Butylamine is one of the four isomeric amines of butane that is known as biomarker of lipid peroxidation (26). However, to our knowledge no direct association between this amine and lipid peroxidation or depression was reported in the literature. However, the dysregulation of butylamine was associated in few works with liver diseases (101).

Additionally, VBAs are produced as a consequence of microbial decarboxylation of amino acids in low pH intestinal tract environment (16, 102). The low pH decarboxylase enzymes act on AAs producing carbon dioxide and free VBAs. Decarboxylase activity was through to be protective mechanism against acid stress across a broad range of naturally occurring acidic circumstances (16). Thus, the downregulation of VBAs in this pilot study could be a potential indicator for low protective mechanism of gut microbiota as response to their environmental change (e.g., pH change) induced by the psychological stressor at awakening through the GBA or due to the chronic and persistent activation of HPA system in rest and in response to short-term exposure to environmental stress in major depression (9).

Altered levels of butane and their derivates such as 2,2-Dimethylbutane have previously been observed in increased amount in exhaled breath of patient with mental disorders, such as schizophrenia and bipolar disorder (34, 35, 103). Butane results from protein oxidation and was considerate as biomarker for lipid peroxidation (26), but the association of 2,2-Dimethylbutane with mental disorders etiology has not been explored.

In summary, the results of our pilot study showed that breath gas analysis by sensitive PTR-MS coupled to advanced machine learning approaches and metabolic pathway analysis can be a powerful tool discriminating MDD patients from HCs with high sensitivity and specificity. Additionally, breathomics offered the possibility for a non-targeted metabolites analysis providing a broad range of altered metabolites and metabolic pathways which were already identified as potential MDD biomarkers in previous bio-fluid and fecal based metabolomics studies that have been linked to the well-known MDD mechanisms including dysregulation of gut microbiome, tryptophan-kynurenine pathway, imbalance of energy metabolism, increased inflammation and oxidative stress and dysfunction of amino acids acting as inhibitory neurotransmitters. On the other hand, the altered VOCs showed decreased responsiveness to awakening stress. This is believed to be related to the impaired functions of the biological systems involved in the gut-brain communication such as HPA.

Despite the aforementioned findings, our study has several limitations. First, the findings of the present study are of correlational nature in a cross-sectional design and thus conclusions about the mechanisms behind MDD cannot be drawn. Moreover, the association between changes in metabolites from breath and metabolites from blood as well brain function has still to be determined. Secondly, the relatively small study population did not allow an accurate analysis of the impact of patient data and habits on the concentration of the metabolites as well categorical comparisons of the MDD subgroups, particularly the difference between treatment outcome groups as well the patient groups with and without medication. We did not observe any significant influence of the medication on the VOCs concentration. However, we cannot rule out that antidepressant drugs could impact the metabolomic profiles. Additionally, smoking was the most important factor in the present study influencing the level of all exhaled VOCs of smoker MDD patients compared to non-smoker ones, but not significantly. However, smoking may enhance oxidative stress through the high concentration of free radicals and oxidants in cigarette smoke or through weakening of the antioxidant defense systems (104). Thus, the impact of smoking habit should be investigated in a larger population in order to rule out that any significant VOCs are due to the oxidative stress enhanced by smoking. In a follow-up study, the sample size needs to be extended for precise predictive performance estimation. We intended thus to recruit 80 patients and 80 controls to investigate the impact of medication, smoking, nutrition, and hormonal status in females on the level of breath metabolites. However, an adequate determination of sample sizes for metabolomic experiments is a complex task due to the unknown nature of the expected effect, the unknown number of the detected metabolites that is highly dependent on the analytical platform, the high-dimensionality, and the strong multi-collinearity of the metabolites. Despite recent efforts, there are currently no standard methods for sample size estimation in metabolomic phenotyping. Third, PTR-MS quadrupole cannot distinguish between substances which contribute to the same mass such as leucine and isoleucine. More sensitive instrumentation such PTR-MS TOF should be used in future investigations to solve the interference problem. The third limitation is related to the low range limit of the used PTR-MS device (< 150). Several amino acids and tryptophan-kynurenine pathway metabolites are slightly volatile and are detectable in a range beyond the range mass limit (~150–200) of PTR-MS. Thus, more sensitive instrumentation with larger masse ranges should be used in the future to detect more compounds in the exhaled breath and determine more disrupted pathways involved in the pathophysiology of MDD. Fourth, only molecules with a proton affinity higher than water can be detected by PTR-MS using water hydronium H_3_O^+^ for the ionization of VOCs. The switching between more precursor ions such as NO^+^ and ${\text{O}}_{2}^{+}$ enables the detection of more compounds and an improved identification of VOCs.

## Data availability statement

The original contributions presented in the study are included in the article/supplementary material, further inquiries can be directed to the corresponding authors.

## Ethics statement

The studies involving human participants were reviewed and approved by Ethics Committee of Medical Faculty of Otto-von-Guericke University Magdeburg. The patients/participants provided their written informed consent to participate in this study.

## Author contributions

LG: acquisition, analysis, interpretation of the data, and drafting the manuscript. TF and CH: conception, design of the study, and interpretation of the data. ML and GM-L: breath samples collection and mass spectrometry profiling with proton transfer reaction mass spectrometry. MF: interpretation of the data and revising the manuscript. All authors contributed to manuscript revision and approved the submitted version.
